# Bibliographical

**Published:** 1887-10

**Authors:** 


### BIBLIOGRAPHICAL.

NINETEENTH CENTURY SENSE. THE PARADOX OF SPIRITUALISM. By JOHN DARBY. Philadelphia: J. B. Lippincott Company. 1887.

It is not probable that we violate any confidence in saying that "John Darby" is the *nom de plume* under which Prof. J. E. Garretson has put forth a number of charming non-professional volumes. The "Seybert Commission," which was the result of a fund left by a wealthy Philadelphian to provide for the expenses of a scientific examination of the claims of spiritualism, is familiar to most reading men, and this volume is in line of the prosecution of that work. It has proved a puzzle to the critics, and few of those to whom it has been sent have attempted anything like a review of it, perhaps because they did not comprehend its scope or fathom its true meaning, possibly, though not probably, because they felt the subject-matter beneath their notice. In any case, the work has not met with the attention which its real merits demand.

There is something strange in the subject of spiritualism, in that it seems to absorb every one who attempts its study. Either sensibly or insensibly they assume a peculiar dreamy, mystical manner when they write of it. They seem to become entirely pervaded by the indefiniteness of their theme. We usually expect that those who have devoted their lives to the investigation of physical matters will become impatient of metaphysics, and demand absolute demonstrations of proposed theories. They usually are realistic, and seldom give themselves up to abstract speculations. It is a matter of surprise, therefore, when we find one like Prof. Garretson attempting to solve a problem that is supposed to be beyond the realm of demonstration. More than that; he, one of the most exact, literal and precise of men, at once becomes recondite, abstruse and enigmatical.

To our apprehension, spiritualism belongs to the domain of the metaphysical. If there be anything in it save mere prestidigitation and sleight of hand, it belongs to the psychological rather than to the somatic school. All through the ages we have had the same outcroppings of the transcendental, appearing under one guise or another, but each one an attempt to comprehend the unfathomable.

Substantially the same speculations have appeared, and hypotheses have been presented differing rather in method than in essence, while the sects founded upon the several dreamings have been known as Gymnosophists in the East and in Africa, as Rosicrucians in Europe, and as Spiritualists in America. There is so much room in metaphysics for all kinds of speculation which it is impossible to disprove that it is little wonder that none of these occult theories have been entirely silenced. It is only when they step over the line which divides the known from the unknown that they are caught. Certain imprudent spiritualists have attempted physical miracles, and then they soon came to grief. As long as they stick to the domain of the unknowable, their assertions are as good as those of any one else, and cannot be disproved. They make mystical affirmations and appeal to the love for study of the marvelous, and in a discussion so familiar to them soon overwhelm the ordinary mind in a maze of unintelligibility. Then they attempt a physical proof, and get caught.

That the impression produced by the book has not met the expectations of its author, is partly the fault of the subject; for it is not the clear, concise and incisive declarations of the master in physics, but it becomes mystical in manner, inconclusive in statement and involved in language. We look for the demonstrations of the mathematician in him; we have the dreaming of the transcendentalist. The subject has overpowered him—it seems out of his domain. That he has proved himself as much a master of the mystical as he had of the positive does not alter the impression, for it seems foreign to him, because we have not known him, save as a physicist. Tyndall may be able to write an excellent novel based upon chivalric times, but for all that it would seem out of place for him, and the most of men would be disappointed with it.

The author of "Nineteenth Century Sense" has chosen a subject that is quite impossible to disprove. If any one were to assert that Mars is peopled with a race of anthropomorphous omniscient mortals, how would an opponent go about to disprove it, and to establish a negative? The proponent would have the advantage by making the first assertion and placing the opponent upon the defensive. If he were to stick to his assertion and simply reason upon it from his own standpoint, he is impregnable. But should he advance to the realm of the known and attempt to establish his hypothesis by finite comparisons and definite illustrations, he takes ground upon which he can successfully be attacked. The spirit of the age is essentially practical, and it has little patience with abstract reasoning. We want what can be demonstrated. *Quod erat demonstrandum* is the summing up of it all.

To the average reader the book reaches its climax on page 40. Yet even that is tantalizing. *What* was the mechanism of the occult? Was the *confrère* the medium in the spiritual, or in the ironical sense? Was he a confederate, or himself deceived? If the whole was but a clever imposture, why the chapters on occult sciences? Was the whole investigation turned into a farce, or is there yet a mystery to be explained, and concerning which we are left in the dark? The book seems unsatisfactory and incomplete. Yet, notwithstanding all, the thoughtful reader will be fascinated with it, and will not be able to lay it down until it is finished. There is such a witchery in vain conjecture about the unknowable; there is such latitude given for metaphysical invention, and there is such enchantment in allowing the rein to fancy, that it is no wonder so many find the greatest charm in Shakespeare's "Tempest," and that the favorite books of children of all growths are Arabian Nights, Robinson Crusoe and Pilgrim's Progress. There is so much room just beyond the borders of the known, that actuality seems dwarfed and circumscribed in comparison.

Yet the very illimitability of the field makes its survey unsatisfactory and unprofitable to the exact mind. There is little to be gained from a disquisition upon the unknowable. Men like to feel the solid rock of demonstration under their feet, and hence, when an author to whom they have looked for substantial facts leads them astray by a phantasm, and presents a *non sequitur* as the summing up of the whole, it is not to be wondered at if they drop all his subtle and ingenious reasonings and turn from his beautiful imagery to more tangible, if less dazzling themes.

We are glad to know that the lack of apprehension of the present work has stimulated the author to the writing of another, which is to be as a ladder leading to it, and which shall have its feet so planted in actuality that there shall be no mistaking its import or its reasoning. In the light of the promised book the first will well be worth a careful study.

